# The molecular diagnostic yield of frame-based stereotactic biopsies in the age of precision neuro-oncology: a cross-sectional study

**DOI:** 10.1007/s00701-023-05742-z

**Published:** 2023-08-09

**Authors:** Obada T. Alhalabi, Felix Sahm, Andreas W. Unterberg, Martin Jakobs

**Affiliations:** 1 Department of Neurosurgery, Heidelberg University Hospital, Im Neuenheimer Feld 400, 69121 Heidelberg, Germany; 2 Department of Neuropathology, Heidelberg University Hospital, Heidelberg, Germany; 3 Division of Stereotactic Neurosurgery, Department of Neurosurgery, Heidelberg University Hospital, Heidelberg, Germany

**Keywords:** Stereotactic biopsy, Glioma, Molecular genetics, Precision oncology

## Abstract

**Purpose:**

With the increasing role of molecular genetics in the diagnostics of intracranial tumors, delivering sufficient representative tissue for such analyses is of paramount importance. This study explored the rate of successful diagnosis after frame-based stereotactic biopsies of intracranial lesions.

**Methods:**

Consecutive patients undergoing frame-based stereotactic biopsies in 2020 and 2021 were included in this retrospective analysis. Cases were classified into three groups: conclusive, diagnosis with missing molecular genetics (MG) data, and inconclusive neuropathological diagnosis.

**Results:**

Of 145 patients, a conclusive diagnosis was possible in *n* = 137 cases (94.5%). For 3 cases (2.0%), diagnosis was established with missing MG data. In 5 cases (3.5%), an inconclusive (tumor) diagnosis was met. Diagnoses comprised mainly WHO 4 glioblastomas (*n* = 73, 56%), CNS lymphomas (*n* = 23, 16%), inflammatory diseases (*n* = 14, 10%), and metastases (*n* = 5, 3%). Methylomics were applied in 49% (*n* = 44) of tumor cases (panel sequencing in *n* = 28, 30% of tumors). The average number of specimens used for MG diagnostics was 5, while the average number of specimens provided was 15. In a univariate analysis, insufficient DNA was associated with an inconclusive diagnosis or a diagnosis with missing MG data (*p* < 0.001). Analyses of planned and implemented trajectories of cases with diagnosis with missing MG data or inconclusive diagnosis (*n* = 8) revealed that regions of interest were reached in almost all cases (*n* = 7).

**Conclusion:**

Although stereotactic frame-based biopsies deliver a limited amount of tissue, they bear high histopathological and molecular genetic diagnostic yields. Given the proven surgical precision of the planned biopsy trajectories, optimizing surveyed lesion regions could help improve the rate of conclusive diagnoses.

## Introduction

Stereotactic biopsies are an established modality for the diagnostic workup of unclear, deep-seated intracranial lesions. This mostly includes lesions suspicious of malignant tumors that cannot be resected in an oncologically sound or functionally safe manner [4]. Paradoxically, being a minimally invasive surgical modality with very low morbidity, the advantage of stereotactic biopsies can at the same time be its very disadvantage. For non-resectable, eloquent, multifocal, and deep-seated intracranial lesions, stereotactic frame-based biopsies can deliver only a finite amount of tissue for neuropathology studies [10]. This can lead to, albeit rarely [6], an inconclusive diagnosis.

While in the past, classic histopathology and immunohistochemistry have been applied in the analysis of biopsy specimen, next-generation sequencing (NGS) and methylation studies have revolutionized the diagnostics of brain tumors in the past. After the updated World Health Organization (WHO) classification of central nervous system (CNS) tumors in 2021, which integrates molecular and histopathological tumor characteristics [11], molecular analyses have indeed become an essential part of the neuropathology workup. In part, this reflects that neuro-oncology is increasingly driven by individualized therapy concepts [2, 27]. However, besides molecular targets, also basic classification often depends on molecular parameters [11]; an emerging role of stereotactic biopsies would not only entail providing sufficient tissue for a viable diagnosis including molecular biomarkers, but also for studies that are translated into personalized targeted therapy for affected patients [5, 11].

In the wake of more complex neuropathology analyses, it would be reasonable to investigate whether the tissue material delivered under current stereotactic biopsy conditions is sufficient to fulfill new diagnostic standards. This study hence aimed to provide an insight into the diagnostic yield of stereotactic frame–based biopsies of intracranial lesions in a high-throughput comprehensive neuro-oncology center with a special focus on tissue molecular genetics. It then explores whether possible surgical factors can be optimized to prevent the delivery of material yielding unsuitable DNA for molecular profiling through stereotactic biopsies and therefore hamper the compilation of an “integrated diagnosis.”

## Methods

In this retrospective study, clinical and histopathological data of all consecutive patients undergoing frame-based stereotactic biopsies for an unclear intracranial lesion in the years 2020 and 2021 at our neurosurgical department were included.

### Surgical procedure

Patients were operated under general anesthesia. After having placed the stereotactic frame, intraoperative computer tomography or magnetic resonance imaging (MRI) imaging was performed to either plan the desired trajectory or fuse it with preoperative images that obtained the stereotactic plan. Biopsies were performed with the stereotactic system (Zamorano-Duchovny or Riechert-Mundinger, inomed Medizintechnik, Emmendingen, Germany) using a guided biopsy forceps (inomed Medizintechnik GmbH, Emmendingen, Germany) via a burr hole trephination placed in line with the trajectory. Specimens were taken in a serial fashion each millimeter from the border of the lesion (labeled *X* mm) to the planned target point within the lesion (labeled ± *0* mm).

### Tissue and image analysis

The overall number of specimen as well as the number used for histological and molecular neuropathology were analyzed. Molecular neuropathological studies included: immunohistochemistry, 850 k methylomics array described under [2], panel sequencing, i.e., customized NGS gene panel capturing the entire coding and selected intronic and promoter regions of 130 genes altered in CNS tumors informing on single nucleotide variations, fusions, and copy number aberrations [21], and RNA sequencing, i.e., next-generation mRNA sequencing [24]. Depending on the final diagnosis in the neuropathology report, cases were assigned to one of the following groups: (1) conclusive, (2) diagnosis with missing molecular genetic data, and (3) inconclusive neuropathological diagnosis.

Pre- and postoperative MRI scans and stereotactic trajectory planning images were analyzed in a subset of cases (inconclusive cases and cases with insufficient isolated DNA for MG). Firstly, post-operative MRI scans were utilized to analyze with trajectories classified into optimal and sub-optimal, depending on their overlap with the region of interest within the tumor (contrast enhancement, fluid attenuated inversion recovery (FLAIR), and necrosis). The positions of numbered specimens were then matched with the specific specimens used for tissues sequencing by the neuropathology department.

### Statistical analysis

Continuous variables are reported as mean ± standard deviation (SD) or median and interquartile range, while ordinal and nominal variables are presented as numbers and frequencies. Comparison of nominal variables between groups was performed using chi-Square or Fisher’s exact test (depending on group size) or non-paired, double-tailed Student’s *t*-test (in paired samples and independent samples). Significance was deemed to be reached at *p* < 0.05 and all statistical analyses were performed using GraphPad PRISM (Version 9; GraphPad Software Inc., Boston, MA; USA).

## Results

### Frame-based stereotactic biopsies bear a sufficient diagnostic yield

In this cohort, a total of 145 consecutive patients undergoing frame-based stereotactic biopsies in 2020 and 2021 as part of a workup for unclear intracranial lesions were included. This cohort (*n* = 80, 55% males) had an average age of 58.3 years (SD ± 20.2 years). Most lesions were deep-seated (thalamus/basal ganglia, 20.1%, corpus callosum 17%) or in eloquent regions of the frontal or temporal lobes (19% and 13%, respectively). Eight percent of the biopsied lesions were infratentorial. Overall, 8% of the patients included showed multifocal lesions. Preoperatively, by means of clinical and radiological features, suspected diagnosis mostly included glioma (66%) and lymphoma (20%). This is also reflected by the radiographic features of these lesions, with contrast enhancement in 85% and FLAIR hyperintensity without contrast enhancement found in the remaining 15% of the cases (see Table 1). Further suspected diagnoses included inflammatory diseases in 8% of the cases and metastasis in 3%. In four cases, pretreated patients were biopsied with the suspicion of tumor recurrence after a tumor board recommendation for tissue sampling with the possibility of personalized therapy.

**Table 1:** Patient characteristics

Patient characteristics	*n* = 145
Age Mean ± SD Median (IQR)	58.3 ± 20.2 61.9 ± 24.6
Gender	Male: *n* = 80 (55%) Female: *n* = 65 (45%)
Locations of lesion Frontal Parietal Temporal Occipital Thalamus/basal ganglia Corpus callosum Infratentorial Multifocal lesions	27 (19%) 9 (6%) 19 (13%) 11 (8%) 31 (21%) 24 (17%) 11 (8%) 12 (8%)
Contrast-enhancing lesions FLAIR lesions	122 (85%) 23 (15%)
Suspected tumor recurrence	*n* = 4 (3%)
Surgical complications	*n* = 2 (1%)
Therapy of malignant entities (*n* = 120) Postoperative chemotherapy Postoperative radiotherapy	*n* = 81/120 (67.5%) *n* = 74/120 (62%)
Glioma cases (*n* = 92) Postoperative off-label therapy	*n* = 4/92 (4%)

*SD* standard deviation, *IQR* interquartile range, *FLAIR* fluid-attenuated inverse recovery

**Fig. 1:**
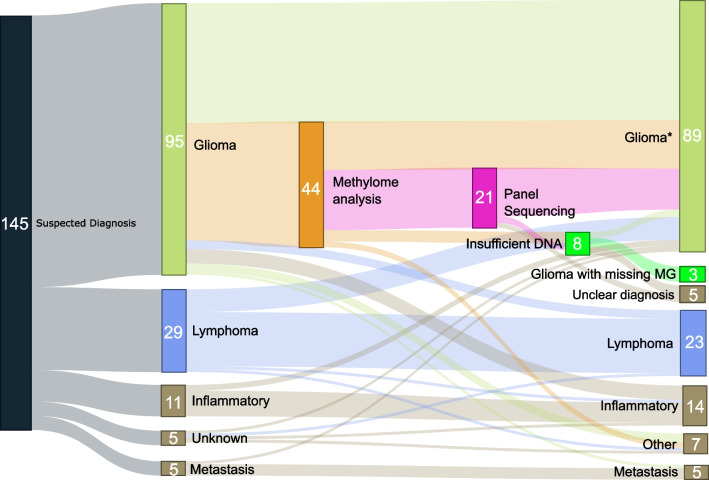
Diagnostic paths of intracranial lesions in the study cohort (*n* = 145). “Other” includes infarction (*n* = 3) and normal tissue (*n* = 4). Glioma* is intended to describe a broad classification of the entity and is not a diagnosis

**Fig. 2:**
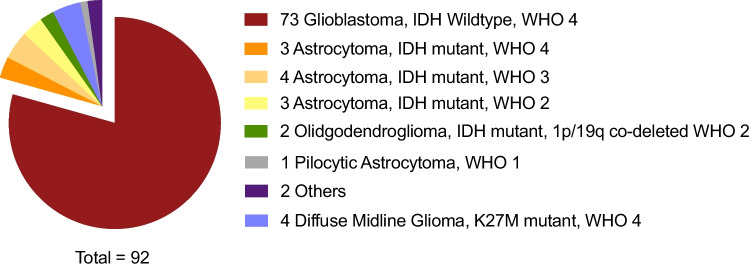
Analysis of pathological diagnoses within the glioma cases. IDH, iso-citrate dehydrogenase; WHO, World’s Health Organization (classification system of CNS tumors); other, glioma, non-otherwise specified (NOS)

After stereotactic frame-based biopsy, the final diagnosis groups in the neuropathology report included glioma in 92 cases (63%), intracranial lymphoma in 23 cases (16%), inflammatory disease in 14 cases, and metastases in five cases (all suspected cases confirmed). A net number of 13 cases (9%) showed a cross-over, i.e., the suspected diagnosis constituted a different entity to the final diagnosis, with most cross-over cases entailing lymphomas to glioma (and less so vice versa, see Figs. 1 and 2).

Most diagnosed glioma cases included isocitrate dehydrogenase (IDH)-wildtype glioblastoma (GBM), WHO grade 4 (*n* = 73, 79% of all gliomas). IDH-mutant astrocytoma was diagnosed in 11 cases (WHO grade 2 to 4), and IDH-mutant, 1p-19q co-deleted oligodendrogliomas in only 2 cases (see Fig. 2). Across all 92 glioma cases, microscopy along with immunohistochemistry and O6-methylguanine-DNA-methyltransferase (MGMT) pyrosequencing was sufficient for a final, integrative diagnosis in 45 cases (49% of the total glioma subset). DNA isolation for methylome analysis was performed in 44 cases (48% of glioma cases). In three of these cases (3% of all diagnosed gliomas), a low amount of DNA was isolated from tissue specimens, whereas in 41 cases, enough DNA was isolated. In a subset of cases where methylation analyses were applied (*n* = 21, 22% of glioma patients), further analyses including next-generation DNA panel sequencing and RNA sequencing (RNA-Seq) were also performed (Fig. 1). From this further subset, only three cases with a low amount of DNA hindered the attainment of a final diagnosis. In total, 89 glioma cases (97%) constituted a complete integrated diagnosis, and only 3 glioma cases remained with missing molecular genetic data due to a low amount of isolated DNA. Two of these cases were then diagnosed as “malignant glioma” (as mentioned in the neuropathology official report). In the third case, BRAF sequencing could not be carried out in an IDH-mutant glioma case, because tumor DNA had already been used up for methylome analysis.

In further 5 cases from the study cohort (3% of all cases), an inconclusive diagnosis was provided. In 3 of the 5 cases, this was attributed the fact that further analyses were not possible due to a low amount of isolated DNA for molecular genetics. In total, eight cases (*n* = 3 glioma with insufficient molecular genetics (MG) and *n* = 5 inconclusive diagnosis) did not show a fully conclusive diagnosis, with insufficient DNA isolated in 6 of these 8 cases. Of note, only one of the 5 inconclusive cases emerged as a GBM in a 3-month follow-up. Interestingly, in 2 cases of conclusive diagnosis (one case of glioblastoma, *IDH*-wildtype, and a further case of inflammatory disease), a disclaimer was issued over quality of the data due to “low DNA amount.” In total, eight samples in the study cohort with a low mount of DNA were identified (see Table 2).

**Table 2:** Comparison of cases in the conclusive group with the other two groups, i.e., cases with glioma diagnosis with missing molecular diagnostics (MG) and inconclusive diagnosis

	Conclusive *n* = 137	Other *n* = 8	*p*-value
Number of cases with insufficient DNA	2 (1%)	6 (75%)	< 0.0001^a^
Radiographic features FLAIR lesions (*n* = 23) Contrast enhancing lesions (*n* = 122)	21 (91%) 116 (95%)	2 (9%) 6 (5%)	0.613^a^
Number of specimens (Standard deviation) provided via biopsy Used for MG analyses	15.76 (4.5) 4.3 (1.1)	15.5 (3.7) 6.4 (2.6)	0.86^b^ < 0.000^b^

^a^Fisher’s exact test

^b^Non-paired, double-tailed Student’s *t*-test

### Cases with inflammatory disease

One case of toxoplasmosis, three cases of CNS vasculitis, two cases of encephalitis, and three further cases of inflammatory demyelinating diseases. In the remaining five cases, CNS tissue with reactive changes was the official final diagnosis.

### Surgical complications

Surgical complications included one non-surgically relevant hemorrhage along the biopsy trajectory, and a single case of surgically revised wound healing disorder, setting the surgical complication rate of stereotactic biopsies in this cohort at 1%.

### Adjuvant therapies

After successful diagnosis of 92 gliomas, 23 lymphomas, and 5 metastases (a total of 120 tumors), 81 (67.5%) patients received chemotherapy and 74 (62%) received postoperative radiotherapy. Of all 92 glioma cases, four patients (4%) received an off-label non-standard of care systemic therapy, based on tissue material from stereotactic biopsies.

## Insufficient DNA material is not a result of a low number of specimens or inaccurate trajectories

To eliminate possible surgical reasons for insufficient DNA material for sequencing or inconclusive diagnoses, we thoroughly examined the previously introduced eight cases. Firstly, we compared the number of specimens delivered in these eight cases with the rest of the patient cohort. Across all patients, an average number of 15.74 specimens were taken (SD, 4.46 specimens). There was no statistically significant difference in number of specimens taken in conclusive (*n* = 138) versus other cases (*n* = 8, glioma with insufficient MG and inconclusive). The average number of specimens in the conclusive group was 15.76 (SD 4.5) vs. an average of 15.5 with a standard deviation of 3.7 in a group containing all other cases (*p* = 0.86, non-paired *t*-test, Fig. 2). As expected, missing DNA was associated with an inconclusive diagnosis (*n* = 3/5) or a diagnosis with missing MG data (*n* = 3/5, total *n* = 6/8) vs *n* = 2/137 in the conclusive diagnosis group (*p* < 0.001, Fisher’s exact test). Because FLAIR lesions are usually less circumscribed on MRI images and can be an expression of a variety of pathologies besides tumors, we determined whether FLAIR lesions were overrepresented in cases without conclusive diagnoses compared to contrast-enhancing lesion. However, differences were non-significant (2/23 FLAIR lesions, *p* = 0.6131, Fisher’s exact test, see Table 2).

**Fig. 3:**
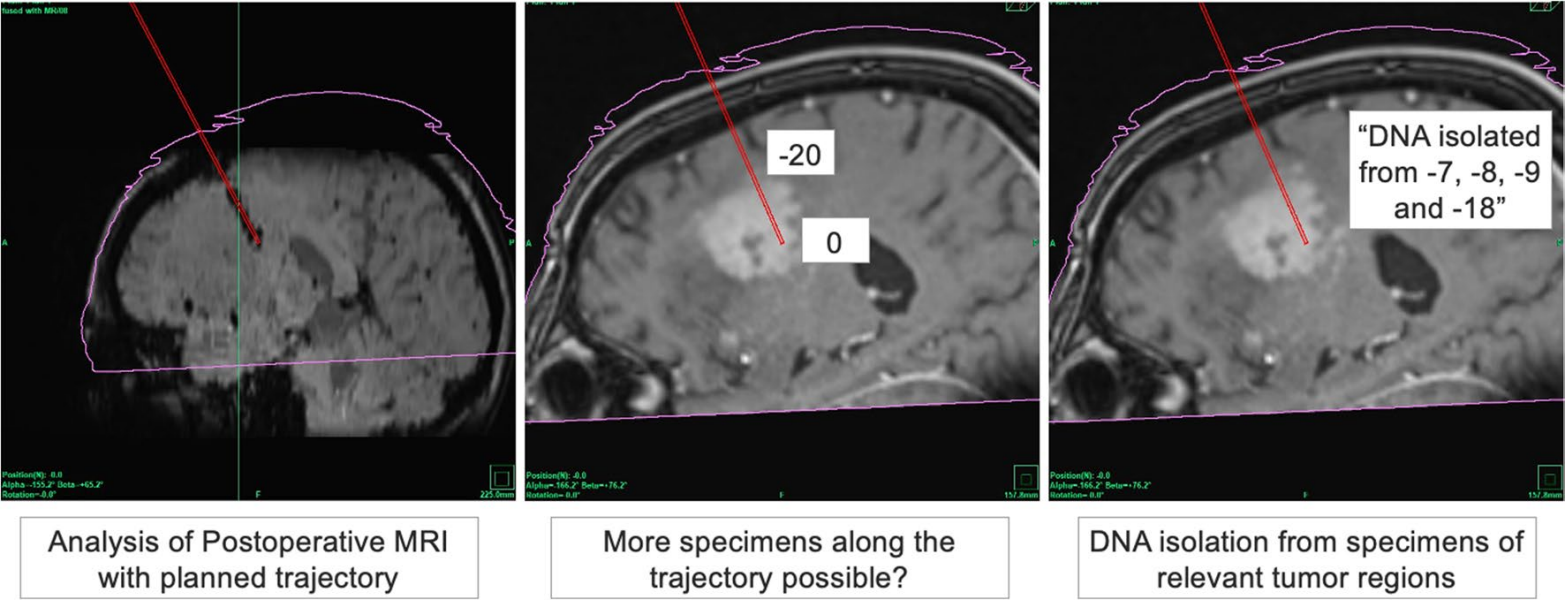
Trajectory analysis of cases with missing MG data and inconclusive diagnosis (*n* = 8). Samples were taken as serial biopsies with each specimen being taken at a 1-mm interval. Specimens were numbered with negative values in relation to their distance in millimeters to the planned final specimen (“0”)

After ruling out that the quantity of delivered specimens was associated with incomplete diagnoses in these 8 cases, we then analyzed the surgically expected “quality” of the specimens. All planned trajectories in these 8 cases were assessed as to (1) whether these trajectories were correctly biopsied based on image fusion of postoperative MRI scans with the planned trajectory (whenever available); (2) in which cases, although potentially less relevant, the number of biopsy specimens could have been maximized based on the radiographic feature of the lesion; and (3) whether the planned trajectories represented the “relevant areas” of the lesion (contrast-enhancement, FLAIR) and how this in turn corresponded to the specimens sequenced by neuropathology (Fig. 3).

In all eight cases, postoperative MRI scans were available and showed that the biopsies were carried out along the planned trajectories (100%). With regard to the number of specimens, in five of the eight cases, more specimens along the planed trajectory could have hypothetically been acquired. The lesions of interested were contrast-enhanced in six cases and FLAIR-hyperintense in two cases. Qualitative analyses of whether the planed trajectories overlap with the region of interest of the lesion revealed that in seven of the eight cases the trajectory was, in opinion of the surgeon performing the biopsy, sufficient to survey the relevant parts of the suspected tumor. In all seven cases with optimal trajectories, specimens used for sequencing analyses originated from the regions of interest. Only in one case (SN 82, see Table 3) the stereotactic trajectory was retrospectively considered nonoptimal (biopsy of mostly the necrotic parts of the lesion), which is why sequencing from the contrast enhancing region of the lesion was not possible.

**Table 3:** Analysis of trajectory for all cases where diagnosis was not “conclusive.” *SN* serial number, *MRI* magnetic resonance imaging, *FLAIR* fluid-attenuated inverse recovery

SN	Correct biopsy of planned trajectory? (postoperative MRI)	More specimens possible?	Evaluation of trajectory	Number of specimens corresponding to tumor regions		
				Contrast	FLAIR	Necrosis
15	Yes	0	OPTIMAL	10	0	4
28	Yes	12	OPTIMAL	0	5	0
46	Yes	3	OPTIMAL	6	0	0
52	Yes	0	OPTIMAL	6	3	0
59	Yes	19	Suboptimal	0	0	8
82	Yes	7	OPTIMAL	20	0	0
86	Yes	4	OPTIMAL	10	0	0
99	Yes	0	OPTIMAL	3	0	0

## Discussion

The increasing complexity of neuropathology reports has been observed by all stakeholders involved in neuro-oncology patient care. In addition to light microscopy and immunohistochemistry, an arsenal of methods including MGMT-pyrosequencing, methylome profiling, panel sequencing, and in rare cases RNA sequencing has become pivotal in providing not only an integrated histopathological and molecular diagnosis, but also further data on tumor epigenetics, transcriptomics, and metabolomics that is becoming increasing relevant to therapy and prognosis. Corresponding to this, the application of targeted tumor therapies is observed in neuro-oncology, either within clinical trials or under off-label conditions especially in progressive or recurrent tumors. At the same time and in frequent cases where tumor recurrence is suspected, ruling out post-therapeutic differential diagnosis like radiation necrosis via stereotactic biopsy surgery could help avoid more extensive forms of respective repeat surgery, especially in patients with tumor-associated reduced status. With this, stereotactic neurosurgery faces the challenge of delivering quantitatively and qualitatively sufficient tissue to meet such expectations [26].

In this analysis, 97% of the cases a sufficient diagnosis was met through stereotactic biopsies. This included the provision of adequate material for analyses beyond what is needed for an “integrated diagnosis.” Indeed, in most cases where isolation of tissue material was needed for methylome profiling, panel sequencing, or RNA sequencing, there was sufficient material to enable this workup. Only in three cases (3% of gliomas), a suspected glioma fell short of an integrated diagnosis due to insufficient DNA material. Overall, in 3% of cases in this cohort, an inconclusive diagnosis was met.

Similar rates for success of stereotactic biopsies in providing a clinically sufficient diagnosis have been reported in previous studies [15, 25]. With molecular data in focus, a study on specifically diffuse intrinsic pontine glioma pediatric patients in 2019 showed lower rates, even when including open biopsies and sub-total resections likely providing more tissue compared to a stereotactic biopsy [18]. At the same time, neuropathological tissue processing methods could be stream-lined at the possible cost of needing more tissue in the future, for example through nano-pore sequencing [14, 17]. In another study with a similar rate of conclusive diagnosis, the rate of applied methylome analyses was lower (44 cases within a 5-year period compared to 44 cases at our center in one year). In a center with neuro-oncology focus, center bias could indeed explain this high rate of methylome studies on unclear intracranial lesions. Therefore, it must be noted that although methylation analyses were performed in these cases, it is unclear in what percentage thereof a final diagnosis would have been possible without such analysis. However, the purpose of the study was to merely demonstrate that such studies, if needed, are almost always feasible under the status quo.

In an interesting observation, a relevant cross-over was observed between suspected intracranial lymphomas and gliomas (and vice versa). In the light of their possible effect on the reliability of the diagnosis [16], delaying admission of corticosteroids until after the biopsy has a direct influence on the clinical management of affected patients, although this notion is currently challenged [22, 23]. Optimization of imaging modalities might help in diminishing this phenomenon [7, 12, 13]. The surgical complication rate in this cohort was, in line with previous studies, very low (< 1%) [20].

In terms of the critical number of specimens for sufficient sequencing workup, this study found that in average, five specimens are needed. This number seems reasonable and feasible, considering the average number of taken specimens in this study (15 to 16, SD of 4). As shown by the data, the number of specimens in general did not present a challenge for conclusive diagnosis, and as expected, a low DNA amount in biopsy probe is associated with an inconclusive diagnosis. The idea that non-contrast enhancing lesions (FLAIR-hyperintense lesions) are “more susceptible” to inconclusive diagnosis was not proven by the data. In the case of FLAIR lesions, established supportive modalities like Positron emission tomography scans are known to improve the visualization of “nodular/cell-rich” and metabolically active regions within the lesion [1, 19].

Only four patients in this cohort underwent stereotactic biopsies for recurrent glial tumors due to progressive disease that were beyond feasible resection options with the specific question of providing tissue material for targeted therapy or ruling out pseudo-progression. The rate of such interventions is projected to increase in the future. The data presented in this analysis, along with similar study results on gliomas and metastases, show that even in this setting, stereotactic biopsies would fulfill expectations [8, 9]. In the wake of the age of precision oncology, it would be reasonable to speculate that more tissue material would be required to fulfill standard of emerging complex neuropathological tests. However, bearing technological advances in mind, it could be equally legitimate to argue that less tumor material is needed due to more efficient diagnostic modalities. According to the findings of the study and in a setting beyond the current state, the concern that “more tissue is needed” than what stereotactic biopsies can provide could not be substantiated. In fact, compared with an earlier study with 500 stereotactic biopsies without MG analysis deeply implemented into the neuropathological workflow, neither the rate of conclusive diagnosis (96.8%) nor the average number of taken specimens (*n* = 16) [3, 28] differed from the results of this analysis. Especially if evaluating stereotactic biopsy against repeat surgery in the setting of a recurrent tumor, a less invasive intervention could be a more viable option, especially with the usually heavily pretreated status of these patients and the peri-operative risk this entails taken into consideration [3, 28].

There are, however, several limitations to this retrospective monocentric study. Firstly, because stereotactic biopsies are known to have an excellent diagnostic yield, an inconclusive diagnosis is a rather rare event. Therefore, a larger patient cohort would have provided more robust data. On the other hand, because the event of DNA isolation was frequent in this cohort (in about 50% of glioma cases), the cohort size is satisfactory and is in general adequate to meet conclusions as to whether in such cases, sufficient DNA material was available. It could be however prudent to study a higher number of patients in order to analyze a larger subset with inconclusive diagnosis and diagnosis with missing MG data, in order to rule out systematic surgical sources of error in a more reliable fashion. Secondly, it is not entirely clear what consequence missing MG data had with respect to the clinical management of affected cases.

In conclusion, although stereotactic frame-based biopsies deliver a finite amount of tissue, they bear an excellent histopathological and molecular genetic diagnostic yield, with rare cases of missing molecular data or rarely inconclusive diagnosis. An optimal trajectory was chosen in almost all inconclusive cases, with DNA isolation from relevant specimens of “relevant” tumor regions. Therefore, none of the surgical variables examined in this study could have been improved to systemically improve the results of this analysis. Therefore, that within the limits of the interpretation of the data presented in this study, in the rare case of insufficient DNA material after stereotactic biopsies, this is not attributable to systematic or surgical aspects.

## Data Availability

Data is available upon reasonable request.
